# 3D-Printed PLA Hollow Microneedles Loaded with Chitosan Nanoparticles for Colorimetric Glucose Detection in Sweat Using Machine Learning

**DOI:** 10.3390/bios15070461

**Published:** 2025-07-18

**Authors:** Anastasia Skonta, Myrto G. Bellou, Haralambos Stamatis

**Affiliations:** Laboratory of Biotechnology, Department of Biological Applications and Technologies, University of Ioannina, 45110 Ioannina, Greece; a.skonta@uoi.gr (A.S.); m.bellou@uoi.gr (M.G.B.)

**Keywords:** chitosan nanoparticles, microneedles, 3D printing, PLA, glucose oxidase, glucose, colorimetric biosensor, machine learning

## Abstract

Biosensors play a central role in the early detection of abnormal glucose levels in individuals with diabetes; therefore, the development of less invasive systems is essential. Herein, a 3D-printed colorimetric biosensor combining microneedles and chitosan nanoparticles was developed for glucose detection in sweat using machine learning. Briefly, hollow 3D-printed polylactic acid microneedles were constructed and loaded with chitosan nanoparticles encapsulating glucose oxidase, horseradish peroxidase, and the chromogenic substrate 2,2′-azino-bis(3-ethylbenzothiazoline-6-sulfonic acid), resulting in the formation of the chitosan nanoparticle−microneedle patches. Glucose detection was performed colorimetrically by first incubating the chitosan nanoparticle−microneedle patches with glucose samples of varying concentrations and then by using photographs of the top side of each microneedle and a color recognition application on a smartphone. The Random Sample Consensus algorithm was used to train a simple linear regression model to predict glucose concentrations in unknown samples. The developed biosensor system exhibited a good linear response range toward glucose (0.025−0.375 mM), a low limit of detection (0.023 mM), a limit of quantification (0.078 mM), high specificity, and recovery rates ranging between 86–112%. Lastly, the biosensor was applied to glucose detection in spiked artificial sweat samples, confirming the potential of the proposed methodology for glucose detection in real samples.

## 1. Introduction

Diabetes mellitus is a prevalent disease in which individuals demonstrate high blood glucose levels [1]. Early detection of abnormal glucose concentrations is critical for timely intervention and regulation, helping to prevent unpleasant symptoms and, in some cases, life-threatening complications [2]. Therefore, glucose serves as a key biomarker that provides essential information for the effective management of diabetes in each individual [3].

Conventional glucose biosensors, also known as blood glucometers, remain the standard option for self-monitoring at home [4]. However, nowadays, less invasive biosensors for glucose monitoring in other biological fluids, such as interstitial fluid, tears, and sweat, are gaining increasing research interest [5]. More specifically, sweat glucose concentration, with a range of 0.00018–20 mg/dL (10 μM–1.1 mM) [6], shows good correlation with blood glucose levels, even though it is almost two orders of magnitude lower [7,8]. Thus, biosensing systems with enhanced analytical performance are of great importance to detect deviations from the physiological range of glucose concentrations, typically between 0.06 and 0.11 mM [9].

To this aim, various classes of biosensors have been developed. Notably, nanozyme-based and other non-enzymatic sensors are well-known for their high stability and sensitivity [10,11]; however, they both lack the superior selectivity exhibited by enzymatic biosensors [12]. This is due to the fact that enzymatic biosensors employ a highly selective enzyme, glucose oxidase (GOx), which catalyzes the oxidation of glucose to gluconic acid and hydrogen peroxide (H_2_O_2_) [13]. Although electrochemical GOx-based biosensors are commonly utilized for glucose detection in sweat [9,14,15], colorimetric biosensors are becoming increasingly popular, as they enable the simple, cost-effective, and real-time monitoring through visible color changes [16,17,18,19,20]. In such systems, a complementary reaction is required to produce the colorimetric signal in response to glucose, such as the oxidation of 2,2′-azino-bis(3-ethylbenzothiazoline-6-sulfonic acid) (ABTS), catalyzed by horseradish peroxidase (HRP) in the presence of H_2_O_2_ generated during glucose oxidation, yielding a blue-green color [21]. To facilitate the process of analyte quantification on a daily basis, smartphones are considered the ideal tools, owing to their portability, real-time image capture, and onboard data analysis [17,18,20].

Chitosan (CS), a polycationic polymer of biological origin, has long been employed in biosensing as an enzyme immobilization carrier due to its unique advantages, including biocompatibility, non-toxicity, enzyme immobilization capacity, porosity (which facilitates analyte diffusion to the biocatalyst), long-term stability, and low cost [22]. Chitosan nanoparticles (CNPs), with sizes in the nanoscale range, possess a high specific surface area, making them ideal for enzyme immobilization and enabling the development of high-performance bioanalytical tools [23]. Enzymes, such as GOx and tyrosinase, have been immobilized on CNPs and applied as biosensing elements in electrochemical biosensors [23,24].

Often in biosensing applications, structural elements known as microneedles (MNs) are employed. As their name suggests, MNs are needle-like structures on the microscale that offer a minimally invasive technique for extracting valuable biomarkers from interstitial fluid [25]. Among MN fabrication methods, three-dimensional (3D) printing has gained considerable traction, as it enables rapid prototyping of MN systems with diverse geometries and morphologies [26]. To date, hollow 3D-printed MNs have been reported for electrochemical pH monitoring in blood [27], as well as for the colorimetric detection of glucose, lactate, and pH [28]. It is worth mentioning that there is a variety of 3D printing techniques; Fused Deposition Modeling (FDM), a technology in which the material in the form of a filament is fed into an extruder and deposited onto the printing bed in a layer-by-layer fashion, is one of the most popular among them [29]. Although FDM is not the first choice when it comes to MN construction due to its lower resolution in contrast to other alternatives, it remains a powerful tool in biomedical applications thanks to the materials that are used [30]. One such material is polylactic acid (PLA), a biopolymer with exceptional properties, such as biocompatibility, biodegradability, and non-toxicity [31], which has already been recruited in MN fabrication [30,32,33,34,35].

The emergence of Artificial Intelligence (AI) and, particularly, machine learning (ML), has paved the way for developing the appropriate models that could rapidly predict the concentration of an analyte of interest on-site based on certain features from large and often complex datasets [36]. Within this framework, both enzymatic and non-enzymatic biosensors have been successfully combined with ML for the estimation of glucose levels in sweat. More specifically, several algorithms have been implemented for this purpose, such as Linear Discriminant Analysis (LDA), Support Vector Machine (SVM), Convolutional Neural Networks (CNN), Decision Tree Regression, and Ensemble Regression algorithms [37,38]. Among the various ML algorithms, Linear Regression (LR) stands out thanks to its simplicity, interpretability, and fast training and predicting speed, making it particularly useful for revealing the linear relationship between features (independent variables) and analytes (dependent variables), particularly helpful in electrochemical and colorimetric biosensors [39,40,41]. The Random Sample Consensus (RANSAC) algorithm is suitable for model training on data containing outliers, as it iteratively selects random subsets, fits a model to each subset, and identifies the model with the highest consensus among inliers [42]. In fact, RANSAC could enhance biosensing, since it offers robust outlier tolerance by excluding inconsistent data points from the final model and enables model fitting in noisy environments [40,43].

In the present work, 3D-printed hollow MNs loaded with CNPs were constructed for glucose biosensing in sweat. First, the hollow MNs were designed and fabricated using FDM technology with PLA filament. During CNP synthesis, GOx, HRP, and the chromogenic substrate ABTS were co-encapsulated, and the hollow MNs were subsequently loaded with the prepared CNPs. Each MN of the biosensing system was subsequently placed in glucose solutions of varying concentrations, and upon contact, the solutions were adsorbed by the CNPs, leading to the oxidation of glucose and ABTS by GOx and HRP, respectively. As a result, a blue-green color developed, which was photographed using a smartphone. Color analysis was conducted either through a smartphone application or computer software. Finally, the RANSAC algorithm was selected to train a linear ML model, which was successfully applied to predict glucose concentration in artificial and real sweat samples. The novelty of this work lies along three main axes: (i) the co-encapsulation of GOx, HRP, and ABTS into CNPs; (ii) the cost-effective construction of 3D-printed hollow PLA MNs that efficiently accommodate the CNPs; and (iii) the ability of each CNP−MN patch to detect glucose concentrations in multiple samples simultaneously. Furthermore, the integration of the biosensor with an ML model enhances the potential applicability of this methodology for non-invasive glucose monitoring.

## 2. Materials and Methods

### 2.1. Materials

Chitosan (low molecular weight, ≥75% deacetylated), GOx by *Aspergillus niger* (135,200 U/g, Type X-S), HRP (150 U/mg), ABTS (≥98%), D(+)-glucose (99.5%), acetic acid (>99.8%), L(+)-lactic acid (>98%), D(−)-fructose, and maltose monohydrate (>95%) were obtained from Sigma-Aldrich (Saint Louis, MO, USA). Urea (>99%) was bought from Fluka (Buchs, Switzerland), and sodium tripolyphosphate (TPP) from Alfa Aesar (Heysham, UK). Sodium chloride (NaCl) was bought from Riedel de Haen (Seelze, Germany), and ammonium chloride (NH_4_Cl) from AppliChem (Darmstadt, Germany). Natural PLA filament (1.75 mm) was purchased from Prima Creator (Malmö, Sweden). The 3D models were printed using the FDM 3D printer Ender 5 from Creality 3D (Shenzhen, China). All solutions were prepared in double-distilled (dd) water, unless stated otherwise.

### 2.2. 3D Design and Printing of PLA MN Patch

**Figure 1:**
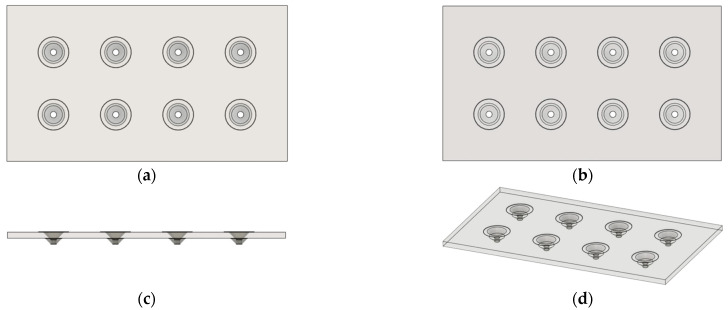
Different views of the final 3D model of the hollow MNs as designed on Autodesk Fusion 360 software. (**a**) Top view; (**b**) Bottom view; (**c**) Front view; (**d**) Angled view.

The 3D design of the MN patch was created using Fusion 360 software v.2.0.21508 provided by Autodesk (San Francisco, CA, USA). Initially, a 36 × 20 × 0.8 mm^3^ rectangular model was designed. Afterward, 8 conical MNs with a radius and a height of 2 mm each and a needle tip hole diameter of 0.4 mm were designed and joined to the initial model. The MNs were evenly spaced and arranged in two parallel rows. Different views of the final model are depicted in Figure 1. The 3D model was then sliced using the Ultimaker Cura 5.2.1 software (Utrecht, The Netherlands). The optimized printing parameters are summarized in Table 1. During slicing, the model was oriented such that the MN base contacted the printing bed (0° orientation), with the needle tips pointing upward, eliminating the need for support structures. Finally, the sliced file was transferred to the 3D printer, and the MNs were printed using natural PLA filament as the printing material and a 0.4 mm brass nozzle (Prima Creator, Malmö, Sweden). The printed models were evaluated for structural integrity and compactness prior to use. The actual printing accuracy of the MN tip inner diameter was measured by using calibrated image analysis in ImageJ 1.54p, based on a high-resolution photograph.

### 2.3. Synthesis of CNPs with Encapsulation of GOx, HRP, and ABTS

The CNPs were synthesized via the ionic gelation method, following a protocol similar to that described by Bellou et al. [44]. Briefly, a 1.5% *w*/*v* CS solution was prepared in 1% *v*/*v* acetic acid, adjusted to pH 5.0, and subsequently diluted to 0.2% *w*/*v* using 50 mM acetate buffer pH 5.0. Stock solutions of GOx, HRP, and ABTS were prepared in 50 mM phosphate buffer at pH 7.0. Appropriate volumes of CS 0.2% *w*/*v* (1 mL), GOx 1.38 mg/mL (13.4 μL), HRP 7.5 mg/mL (29.6 μL), and ABTS 20 mM (37 μL) were mixed to reach a final volume of 1.08 mL. The mixture was subsequently incubated in a thermomixer at 30 °C and 850 rpm for 10 min. Subsequently, 400 μL of sodium tripolyphosphate (TPP, 2.5 mg/mL) was added dropwise to the CS−GOx−HRP−ABTS mixture, yielding a turbid suspension. The final concentrations of GOx, HRP, and ABTS were equal to 12.5 μg/mL, 150 μg/mL, and 0.5 mM, respectively, with a CS/TPP *w*/*w* ratio of 2:1. The mixture was further incubated at 30 °C and 850 rpm for 30 min, then centrifuged at 14,000 rpm for 20 min. The supernatant was discarded, whereas the precipitate was washed twice with 50 mM phosphate buffer at pH 7.0. Finally, the precipitate was lyophilized, powdered, and stored at −20 °C until further use. For the synthesis of neat CNPs, the same methodology was followed, substituting the GOx solution with phosphate buffer.

The encapsulation efficiency (EE (%)) of the enzymes was determined using CNPs that co-encapsulated GOx and HRP, in the absence of ABTS, according to Equation (1):

(1) $$EE(\%)=\frac{U_{0}-(U_{sup}+U_{w1}+U_{w2})}{U_{0}}\times 100$$

where U_0_ is the initial rate (ΔA/min) of the GOx or HRP solution before co-encapsulation, U_sup_ is the initial rate (ΔA/min) measured in the supernatant of the first centrifugation after GOx or HRP co-encapsulation, and U_w1_ and U_w2_ are the initial rates (ΔA/min) in the first and second washing solutions of the CNPs, respectively.

The initial rate (ΔA/min) of GOx was determined by incubating GOx (0.5 μg/mL), HRP (20 μg/mL), ABTS (2 mM), and glucose (2.5 mM) in a final volume of 200 μL at pH 7.0 and 30 °C for 5 min. The initial rate (ΔA/min) of HRP was determined by incubating HRP (0.4 μg/mL), ABTS (0.5 mM), and H_2_O_2_ (0.1 mM) in a final volume of 200 μL at pH 7.0 and 30 °C for 10 min. In both assays, the absorbance of the solutions was measured at 405 nm.

### 2.4. Fabrication of CNP−MN Patch Biosensor

To fabricate the CNP−MN patch biosensor, 1 mg of CNPs was accurately weighed on aluminum foil using an analytical microbalance. The weighed amount of nanomaterial was then carefully transferred into a hollow MN using a weighing spatula. This procedure was repeated until all MNs on a single MN patch were filled with CNPs.

### 2.5. Optimization of Glucose Detection

To determine the optimal time required for the CNPs to develop a stable color response, the MNs were incubated with solutions of artificial sweat containing glucose concentrations ranging from 0.1 to 1 mM. Photographs were taken at 5 min intervals, up to a total of 20 min. The mean volume of a liquid sample that could be adsorbed by the CNPs was calculated after incubating 1 mg of CNPs with 200 μL of dd water for 10 min, followed by centrifugation at 14,000 rpm for 20 min. The supernatant was discarded, and the CNPs were weighed again to calculate the adsorbed volume. All experiments were performed in triplicate.

### 2.6. Glucose Determination Using CNP−MN Patch Biosensor in Artificial Sweat

Glucose detection by the CNP−MN patch is based on the enzymatic reactions catalyzed by GOx and HRP in response to the presence of glucose, as described in Equations (2) and (3). Since the biosensor incorporates GOx, HRP, and ABTS, it can be directly applied to glucose-containing solutions to estimate the concentration of the biomarker. Higher glucose levels yield more ABTS^+^ and, consequently, a more intense green color.

(2) $$Glucose+O_{2}\;\overset{GOx}{\to }Gluconic\;acid+H_{2}O_{2}$$

(3) $$ABTS+H_{2}O_{2}\;\overset{HRP}{\to }\;{ABTS}^{+}+H_{2}O$$

Artificial sweat was prepared as in Xiao et al. [17] by dissolving 20 g/L NaCl, 17.5 g/L NH_4_Cl, 5 g/L acetic acid, and 15 g/L lactic acid in dd water. The pH of artificial sweat was adjusted to 6.0 by adding an appropriate amount of NaOH 2 M. To determine glucose concentration in artificial sweat using the CNP−MN patch biosensor, glucose solutions of varying concentrations (0.025–1 mM) were prepared in artificial sweat. Then, 5 μL of either neat artificial sweat or glucose-containing artificial sweat was dropped onto each MN and incubated for 10 min at room temperature (RT). The base of the MNs was photographed using a smartphone (Samsung Galaxy A55, Samsung India Electronics Pvt. Ltd., New Delhi, India) under standardized lighting conditions and in the presence of 3 photographic reference cards (JJC 3in1 Digital Gray Card and White Balance (GC-2)) for subsequent color correction. The color correction of the photographs was performed using the open-source image editing software GIMP (version 2.10.32). The corrected photographs were then analyzed either in Color Grab application or in ImageJ software to extract the L, a, and b colorimetric parameters from the CIELAB color space by creating circular regions of interest (ROIs) for each sample. The ΔΕ values were calculated using Equation (4), as described by Xiao et al. [16]:

(4) $$\Delta E=\sqrt{{\left(L-L^{\prime }\right)}^{2}+{\left(a-a^{\prime }\right)}^{2}+{\left(b-b^{\prime }\right)}^{2}}$$

where L, a, and b are the lightness, the red/green coordinate, and the yellow/blue coordinate, respectively, in the absence of glucose, and L′, a′, and b′ represent the same parameters in the presence of glucose after its enzymatic oxidation. All experiments were conducted in triplicate.

### 2.7. Reproducibility and Storage Stability of GOx−HRP−ABTS CNPs

The reproducibility of the GOx−HRP−ABTS CNPs was assessed by incubating 5 MNs with 0.4 mM glucose solution in artificial sweat, and 1 MN with neat artificial sweat as a negative control, at RT for 10 min. The results were expressed as relative standard deviation (RSD).

The storage stability of the biosensor was studied by storing the CNPs at −20 °C, 4 °C, and RT for 3 weeks. Each week, the remaining ΔΕ (%) was calculated by incubating the CNP−loaded MNs with 0.4 mM glucose solution in artificial sweat, considering the initially measured ΔΕ at t = 0 as the 100% reference value.

### 2.8. Analytical Performance of the Biosensor

The analytical performance of the biosensor was studied in terms of its linear range, LOD, LOQ, sensitivity, and specificity. The linear response range toward glucose was determined as described in Section 2.6 by constructing a calibration curve of glucose versus ΔΕ. The LOD and LOQ of the biosensor were calculated based on the standard deviation of the ΔΕ values from blank samples and the slope of the calibration curve, as described in [21]. Sensitivity was calculated from the slope of the same calibration curve. The specificity of the biosensor toward glucose was investigated by comparing the color response of the CNP−MN patch after 10 min incubation with glucose, fructose, maltose, and urea, each at a concentration of 0.4 mM.

### 2.9. Machine Learning for Glucose Prediction in Sweat

A linear regression model was trained to evaluate its suitability for estimating glucose concentrations in new, unknown samples. A dataset of 109 samples, spanning a glucose concentration range of 0.087−0.375 mM, was created to train the model using a single feature, ΔΕ. The ML model was implemented in Python 3.11.13 using Google Colab, with the following libraries: Pandas 2.2.2, Scikit-learn 1.6.1, NumPy 2.0.2, Matplotlib 3.10.0, and SciPy 1.15.3. Generative AI (ChatGPT, GPT-4o, OpenAI) was occasionally used to assist with code syntax and implementation during model development. The dataset was first split into 2 subsets—a training and a test set—at an 80:20 ratio, with the random state set to 0 to ensure reproducibility. A simple linear regression model was then trained using the RANSAC algorithm, also with the random state fixed at 0 and all other parameters left at their default values. Model performance was assessed by applying the trained model to the test set and calculating performance metrics, including the coefficient of determination (R^2^), mean absolute error (MAE), and root mean squared error (RMSE). A parity plot comparing actual and predicted glucose concentrations was also generated. In addition, 5-fold cross-validation was performed across the dataset to compute average R^2^, MAE, and RMSE values.

In detail, model accuracy was determined by R^2^, which indicates how well the model fits the data. The values of R^2^ range between 0 and 1, with higher values reflecting a better fit and greater predictive accuracy [45]. However, in some studies, it may be reported as a percentage (0−100%) [37,46]. The MAE represents the average of the absolute differences between the actual (yi) and predicted (ŷi) values in the test set (Equation (5)), while RMSE is defined as the square root of the mean squared differences between predicted and actual values (Equation (6)). The lower the values of these metrics, the higher the predictive accuracy of the model [45].

(5) $$MAE=\frac{1}{n}\sum_{i=1}^{n}\left|yi-ŷi\right|$$

(6) $$RMSE=\sqrt{\frac{\;\sum_{i=1}^{n}{\left(yi-ŷi\right)}^{2}}{n}}$$

### 2.10. Spiking Study in Artificial Sweat

Following model development, a spiking study was performed in artificial sweat to evaluate whether the proposed biosensing methodology could serve as an accurate alternative for glucose detection in sweat. To this aim, glucose was added to artificial sweat to achieve final concentrations of 0.1, 0.2, and 0.375 mM. The procedure for glucose determination was the same as that described in Section 2.6. The corresponding ΔE values were then used as inputs to the trained ML model to predict the glucose concentrations of the spiked samples. The recovery rate of spiked glucose was calculated using Equation (7). The experiment was conducted in triplicate.

(7) $$R(\%)=\frac{spiked\;sample\;result-unspiked\;sample\;result}{known\;spike\;added\;concentration}\times 100\%$$

### 2.11. Glucose Determination in Real Sweat

Sweat samples were collected from a healthy female volunteer (27 years old) after obtaining informed consent. The first sweat sample was collected from the forehead of the volunteer following 45 min of exercise, without prior glucose intake. The second sample was obtained after consumption of 30 g glucose, 30 min prior to exercise. Both samples were applied to the CNP–MN patch as described in Section 2.6, and glucose concentrations were predicted using the trained ML model.

## 3. Results and Discussion

**Figure 2:**
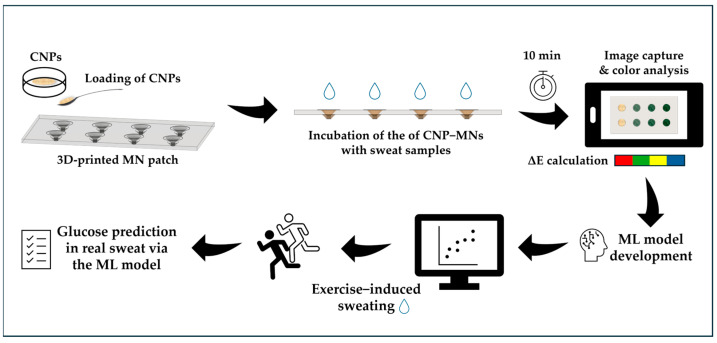
Workflow of the CNP–MN colorimetric biosensor for glucose detection in sweat.

In the present study, a colorimetric glucose biosensor based on CNPs and 3D-printed hollow PLA MNs was developed for glucose detection in sweat, using a smartphone and an ML model (Figure 2). Upon incubation with sweat samples, the CNPs developed a green color, the intensity of which depended on the glucose concentration. A smartphone was then employed for both image capture and color analysis. This approach enabled the construction of a dataset linking color differences (ΔΕ) to glucose concentrations, which was used to train a linear ML model via the RANSAC algorithm. The trained model could then be used to predict glucose levels in unknown samples. The combined use of CNP−MN patches, smartphone technology, and ML offers a fast, accessible, and user−friendly strategy for non-invasive glucose detection in sweat.

### 3.1. Optimization of Biosensor Construction

**Figure 3:**
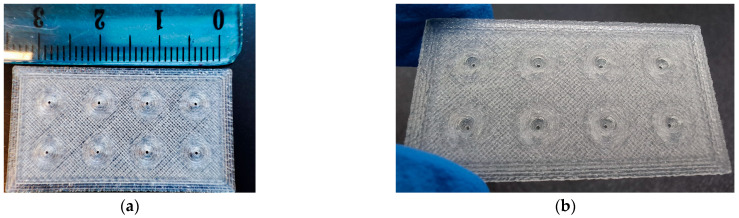
Photographs of a 3D-printed hollow PLA MN patch. (**a**) Top view; (**b**) Bottom view.

The developed biosensor consists of 2 main components: the 3D-printed hollow MNs and the CNPs. The 3D printing process was optimized to efficiently accommodate the formed CNPs. The flexibility provided by this technology allowed for the customization of the model according to the specific research needs. Two main models were created for this work: one with a capacity of 2 mg of nanomaterial and another with a capacity of 1 mg. The second was preferred because it used only half the amount of CNPs to fill each MN, making the whole system more sustainable. Moreover, the 3D-printed system could be reused, contributing even more to the overall sustainability. The diameter of the hole on the tip of the needles was set to 0.4 mm, as it retained the CNPs in the MN cavities and allowed for liquid adsorption. It was confirmed that the actual printing accuracy of the MN tip inner diameter was 0.44 ± 0.04 mm. The number of needles per MN patch was also adjusted, so models with 4, 6, and 8 needles were constructed. A 3D-printed MN patch with 8 needles is illustrated in Figure 3.

Regarding CNP synthesis, the CS and TPP concentrations, as well as the CS/TPP *w*/*w* ratio, were selected based on our previous work [44]. The incorporation of GOx, HRP, and ABTS took place during CNP formation, following the approach of Wang and Jiang [47], rather than incubating the formed NPs with a mixture of the 3 components afterward as proposed in other studies [24,48], based on preliminary experiments using only GOx. The optimal concentration of GOx was selected based on preliminary results showing that, at 12.5 μg/mL, the biosensor exhibited a clear response to glucose concentrations in the range of 100–200 μM. However, when lower or higher GOx concentrations were used, no improvement was observed; in some cases, the color change was even reduced. The optimal concentrations of HRP and ABTS were determined in a similar manner and set to 150 μg/mL and 0.5 mM, respectively. The EE (%) of GOx and HRP following co-encapsulation was determined to be 59% and 26%, respectively.

An important advantage of the present approach is the potential to simultaneously detect multiple analytes in sweat without the risk of contamination, since each needle could contain CNPs formulated to respond to a specific biomarker, inspired by the works of He et al. [49] and Yue et al. [19]. Thus, the design flexibility offered by 3D printing, through the ability to create MNs with various geometries and configurations, along with the possibility to encapsulate different sensing components in CNPs, offers a valuable platform for non-invasive biosensing. Up until now, 3D-printed solid PLA MNs have been proposed for transdermal drug delivery [30,33,34]; however, their application in biosensing remains largely unexplored. Additionally, 3D-printed hollow PLA MNs were fabricated elsewhere [35] using a method that involved chemical etching to create the needle-tip opening, which was found to be inefficient yet promising for further investigation.

### 3.2. Optimization of Glucose Detection

**Figure 4:**
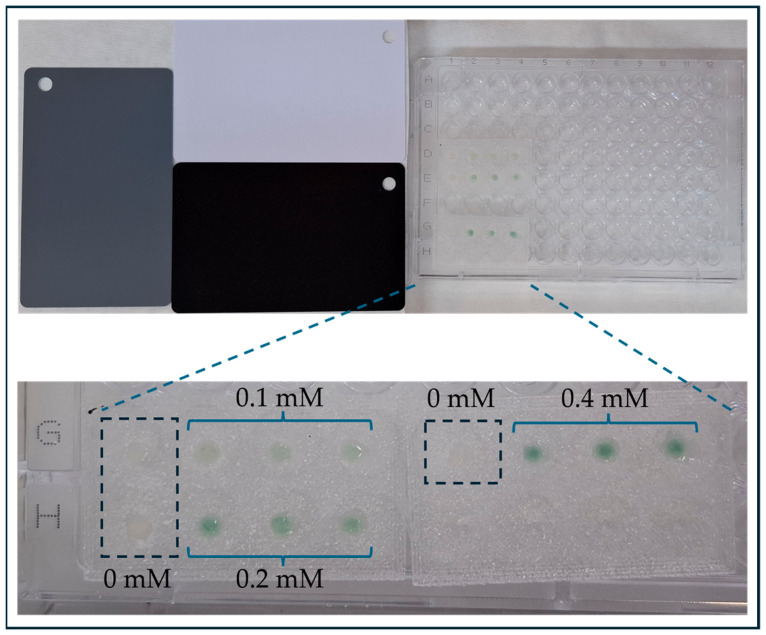
Experimental setup for glucose detection in artificial sweat using CNP–MN patches. Color reference cards were included for image normalization and to minimize the effect of lighting variability. Dotted lines indicate magnified views of the patches. Glucose concentrations are labeled (0, 0.1, 0.2, and 0.4 mM).

For glucose detection, the CNP−MN patch was placed in artificial sweat samples either with or without glucose. Through the openings at the needle tips, CNPs adsorbed sweat, initiating the enzymatic cascade between glucose, GOx, HRP, and ABTS, which led to a visible color change in the nanoparticles from white/pale yellow to green (Figure 4). A smartphone was then used to capture color changes of the samples on the basis of the MNs, and the L, a, and b color parameters were extracted either via a color recognition application or using ImageJ software for batch analysis. To ensure consistent detection under varying lighting conditions, color reference cards were included in each image, and photographs were normalized before analysis. Color difference (ΔΕ) was subsequently correlated with glucose concentrations. The selection of this color parameter over others was based on the linear relationship observed between ΔΕ values and glucose concentrations, characterized by a high R^2^ (0.99). Additionally, CIELAB color space is less sensitive to illumination variability compared to RGB [50], which is an important feature for a biosensing system relying on smartphone-based image capture under real-world conditions.

**Figure 5:**
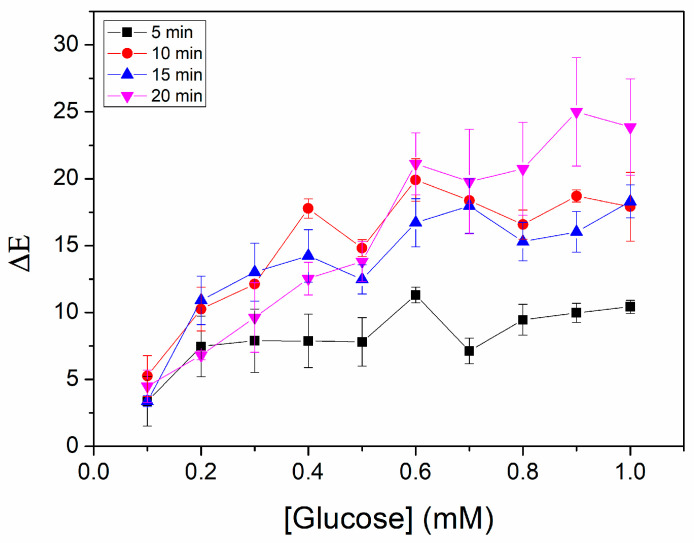
Optimization of the reaction time for ΔΕ stabilization.

The detection of glucose was optimized in terms of the time required for the CNPs to develop a stable color and the sample volume needed for the CNPs to become saturated with water. The optimal reaction time was determined to be 10 min, as can be concluded from Figure 5, since ΔΕ values plateau after this point for glucose concentrations above 0.3 mM. Moreover, the mean volume of artificial sweat that could be adsorbed by the CNPs was calculated to be 4.94 ± 0.25 μL. The reaction time reported here is improved in comparison to that of Xiao et al. [16], where ΔE required 15 min to reach stable values. The reported sample volume in the same study (4.15 μL) is lower, although comparable, to ours. A comprehensive list of recent advances in the field of colorimetric glucose biosensors for sweat analysis, including a comparison of their characteristics, is presented in Table 2.

### 3.3. Reproducibility and Storage Stability of GOx−HRP−ABTS CNPs

**Figure 6:**
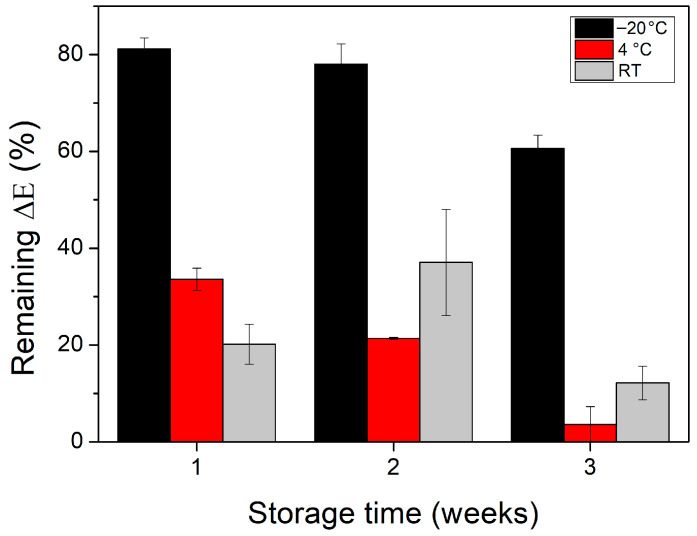
Storage stability of GOx−HRP−ABTS CNPs at −20 °C, 4 °C, and RT.

The optimized CNP−MN patch biosensor was evaluated under the optimized reaction conditions for both reproducibility and storage stability. The developed biosensor exhibited good reproducibility, with an RSD of 3.90%. The storage stability of the biosensor was studied at −20 °C, 4 °C, and RT over a period of 3 weeks. The relative ΔΕ (%) of CNPs at each storage temperature is presented in Figure 6. When stored at 4 °C and RT, the biosensor lost approximately 66% and 80%, respectively, of its initial color response after just 1 week of storage. A similar trend was observed in a previous study, where GOx non-covalently immobilized onto CNPs and stored at 4 °C retained ~90% activity after 7 days, with a further 10–15% loss over the following 3 days [24]. The phenomenon of the significantly reduced stability of the CNP−MN system may be attributed to HRP, which is known to be less robust than GOx [51]. On the other hand, when the CNP−MN system was stored at −20 °C, it was characterized by markedly improved stability, retaining almost 80% and 60% of its initial color response after 2 and 3 weeks of storage, respectively. The requirement for storage at −20 °C could represent a limitation of this biosensing system. Overall, the incorporation of coupling agents [52,53], additives [54,55], or/and ionic liquids [56] during enzyme immobilization could potentially lead to enhanced results by exerting a long-term stabilizing effect on the enzymes.

### 3.4. Specificity Study of the Biosensor

**Figure 7:**
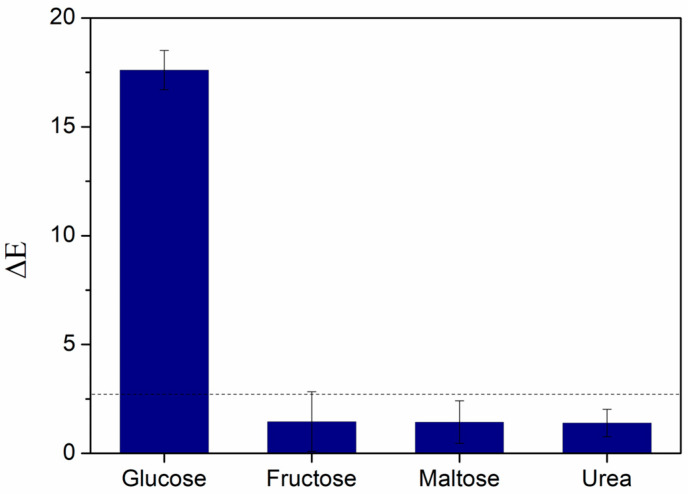
Specificity study of the CNP−MN biosensor toward glucose, fructose, maltose, and urea. The dotted line indicates the lower ΔE threshold above which glucose levels are considered detectable.

The CNP−MN biosensor was further tested for its specificity toward glucose. To assess this property, each needle was incubated with a different potential interferent commonly found in sweat and, more specifically, with glucose, fructose, maltose, and urea, all at an equal concentration of 0.4 mM. The biosensor responded specifically to glucose, while the colorimetric responses to fructose, maltose, and urea were insignificant, with ΔE values below detectable levels (Figure 7). Therefore, the CNP–MN biosensor is suitable for detecting this biomarker in complex biological matrices such as sweat.

### 3.5. Analytical Performance of the Biosensor

**Figure 8:**
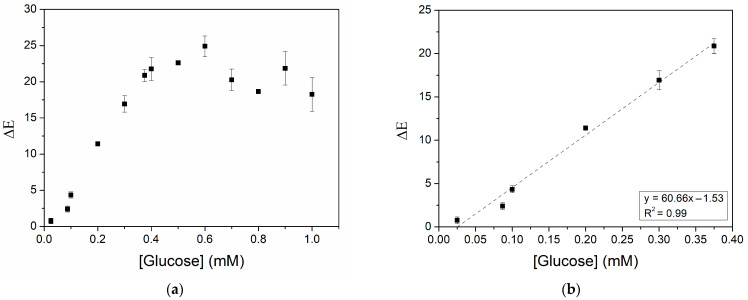
Colorimetric response of the CNP−MN biosensor to varying glucose concentrations: (**a**) 0.025−1 mM; (**b**) 0.025−0.375 mM (linear range).

Another aspect of the biosensor that was investigated was its analytical performance, namely the linear response range, LOD, LOQ, and sensitivity of the CNP−MN system toward glucose. Artificial sweat samples containing varying glucose concentrations in the range of 0.025–1 mM (Figure 8a) were used, and a calibration curve of glucose was constructed (Figure 8b). The response of the colorimetric biosensor to glucose was linear between 0.025 mM and 0.375 mM, the LOD was 0.023 mM, and the LOQ was 0.078 mM. The sensitivity of the system was calculated as 60.66 mM^−1^. Since physiological glucose levels in sweat typically range between 0.06 and 0.11 mΜ [9], the proposed biosensor can effectively detect physiological, as well as hypoglycemic and hyperglycemic, glucose concentrations—specifically in the range of 0.025 to 0.375 mM. Nonetheless, a limitation of the CNP−MN biosensor is its inability to quantify glucose levels below 0.078 mM. Comparing the performance of the present biosensor with other colorimetric glucose biosensors for sweat detection (Table 2), it is evident that the lower limit of the linear range in our study (0.025 mΜ) is improved in contrast to that of Xiao et al. (0.1 mΜ) [16], Xiao et al. (0.05 mΜ) [17], and Zhou et al. (0.03 mM) [37], while the upper limit (0.375 mΜ) exceeds that reported by Xiao et al. (0.25 mΜ) [17]. Furthermore, the LOD reported here is lower than that of the majority of comparable studies [16,17,19,37]. These findings support the potential applicability of the CNP−MN patch biosensor for colorimetric glucose monitoring in sweat. It is also noteworthy that covalent enzyme immobilization could enhance not only storage stability, as previously discussed, but also sensitivity, compared to non-covalent immobilization methods [57]. Therefore, future studies could investigate the impact of covalent immobilization on the analytical performance of the biosensor.

### 3.6. Machine Learning for Glucose Prediction in Sweat

**Figure 9:**
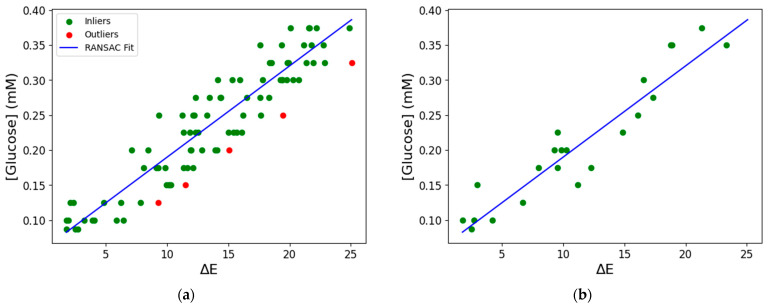
Fitted RANSAC regression model of ΔΕ against glucose concentration on: (**a**) Training set; (**b**) Test set.

**Figure 10:**
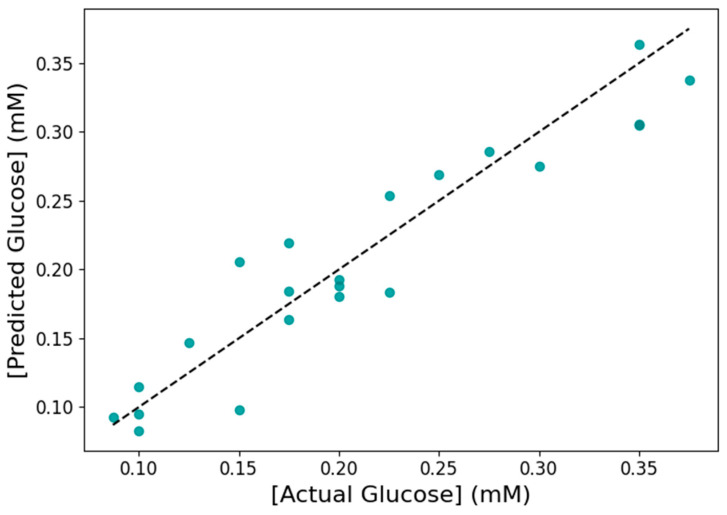
Parity plot of the predicted and actual glucose concentrations for the test set.

The applicability of ML for predicting glucose levels in sweat based on the corresponding ΔΕ values was investigated next. First, the presence of a strong positive linear correlation between ΔΕ and glucose concentration was confirmed by Pearson’s correlation coefficient (r = 0.93, *p* < 0.05). Based on this finding, a simple linear regression model was considered an appropriate choice for the evaluation of glucose concentration in the range 0.087–0.375 mM, using ΔΕ as the predictor variable. For this purpose, the RANSAC algorithm was selected for model development, as it is a robust tool for outlier detection. The model was initially trained using 80% of the dataset (n = 87 samples). As shown in Figure 9a, the algorithm fitted a linear model based on 82 data points, excluding 5 outliers. The model was then applied to the test set (Figure 9b), and its performance was evaluated using both the test set (n = 22 samples) and 5-fold cross-validation on the full dataset, with R^2^, MAE, and RMSE as performance metrics (Table 3). Regarding the test set, the model achieved an R^2^ of 0.89, an MAE of 0.0245 mM, and an RMSE of 0.0292 mM. The corresponding average values from 5-fold cross-validation were R^2^ = 0.85 ± 0.02, MAE = 0.0267 ± 0.0015 mM, and RMSE = 0.0324 ± 0.0022 mM. In a related study, the accuracy of an SVM model for a colorimetric TMB−based glucose biosensor in artificial sweat, obtained after 5-fold cross-validation, was 0.910 and 0.951 when 3 and 15 features were used, respectively [37]. Although slightly less accurate, the model developed in the present study is simpler, more interpretable, and still demonstrates a good predictive capacity. The suitability of a linear model for glucose detection was also highlighted in Poddar et al. [46]. The precision of the model presented here was further evaluated using a parity plot comparing the predicted and actual glucose concentrations in the test set (Figure 10). The close alignment of the predicted values with the actual ones, as indicated by the dotted line, reinforces the applicability of the model for glucose prediction in sweat. These results demonstrate that the model is highly accurate and generalizable, supporting its potential use for estimating glucose levels in unknown samples under real conditions.

### 3.7. Spiking Study Using CNP−MN Biosensor and ML

The accuracy of the proposed methodology for glucose monitoring in sweat, combining the CNP–MN biosensor and the trained linear model, was assessed by incubating the biosensor with 3 distinct glucose concentrations (0.100, 0.200, and 0.375 mM) in artificial sweat. The predicted glucose concentrations were equal to 0.110, 0.180, and 0.320 mM for artificial sweat solutions spiked with 0.100, 0.200, and 0.375 mM of glucose, respectively (Table 4). The corresponding recovery rates were 112 ± 3%, 89 ± 2%, and 86 ± 1%, a result that highlights the high accuracy of the developed biosensor and the predictive capacity of the ML model.

### 3.8. Application in Real Sweat Sample Using CNP−MNs Biosensor and ML

The feasibility of the developed biosensing system for detecting glucose levels was further investigated using real sweat samples collected from a healthy volunteer, both before and after glucose consumption. Sweat was collected from the forehead of the volunteer following 45 min of exercise and then incubated with the CNP−MN biosensor. For each sample, ΔΕ values were extracted and input into the trained model to predict the corresponding glucose concentration in sweat. When no glucose was consumed, the biomarker concentration in sweat was apparently below the LOQ, so no glucose was detected by the biosensor. However, after the intake of 30 g glucose, the biomarker was detected in sweat, with a predicted concentration of 0.103 ± 0.002 mM. As a control, CNPs lacking GOx were also incubated with the sweat samples to confirm that the observed color change resulted specifically from enzymatic glucose oxidation. These results suggest that both the CNP−MN biosensor and the trained ML model can be applied for glucose estimation in real sweat. In future studies, the incorporation of a compound that would induce sweating at rest, such as pilocarpine, could further extend the applicability of the biosensor for real-time and on-demand glucose monitoring.

## 4. Conclusions

In summary, the present study demonstrates that enzymes such as GOx and HRP, along with the chromogenic substrate ABTS, can be encapsulated into CNPs, yielding a glucose-responsive colorimetric biosensing element. The construction of 3D-printed PLA hollow MNs provided a platform for loading the CNPs, which could then adsorb sweat through tip openings. In the presence of glucose, a visible color change occurred, and a smartphone was used to monitor glucose levels by capturing color differences, even under varying light conditions, thanks to image normalization with reference cards. To support the practical application of the system, a simple linear regression model was developed for predicting glucose concentrations in artificial sweat. Overall, the biosensor demonstrated a response time of 10 min, required only 5 μL of sample, and exhibited good analytical performance, with a low LOD (0.023 mM), LOQ (0.078 mM), and a linear response range toward glucose (0.025–0.375 mM). In addition, the biosensor proved to be highly specific to glucose and relatively stable when stored at −20 °C. The developed linear model was sufficiently accurate, with an R^2^ of 0.85 ± 0.02, MAE of 0.0267 ± 0.0015 mM, and RMSE of 0.0324 ± 0.0022 mM, based on 5-fold cross-validation. The validity of the proposed methodology for glucose prediction was further supported by a spiking study in artificial sweat and successful glucose detection in real sweat.

The proposed biosensor offers several advantages in terms of non-invasiveness and potential for integration into wearable health monitoring systems. More specifically, this biosensor enables cost-effective glucose detection through sweat, eliminating the need for blood sampling. Current efforts are directed toward enabling the biosensor to simultaneously detect multiple analytes of interest in sweat upon contact with skin. To support broader usability, a smartphone application enabling user-friendly, on-site prediction of analyte levels directly from photographs could also be developed.

## Figures and Tables

**Table 1:** Printing parameters of MN patches in the slicing software.

Printing Parameter	Set Value
Layer height	0.12 mm
Infill density	100%
Printing temperature	200 °C
Build plate temperature	50 °C
Print speed	80 mm/s

**Table 2:** Recent advances in glucose colorimetric biosensors for application in sweat.

Immobilization Support	Detection System	Color Space/Parameter	Results	Ref.
Whatman filter paper	GOx–HRP–o-dianisidine	CIELAB/ΔΕ	Reaction time: 15 min Sample volume: 4.15 μL Linear range: 0.1−0.5 mM LOD: 0.03 mM	[16]
Filter paper/CS	GOx−HRP−TMB ^1^	RGB/R ^2^	Reaction time: 10 min Sample volume: 23.8 ± 1.1 μL Linear range: 50–250 μM LOD: ~35 μΜ Sensitivity: −0.19 μM^−1^	[17]
Filter paper	GOx−HRP−TMB	Pixel intensity	Reaction time: 3 min Sample volume: 3 μL Linear range: 0.01–0.15 mM LOD: 0.01 mM	[18]
Whatman filter paper/CS	GOx−KI	RGB/G	Sample volume: 2.5 μL Linear range: 0–2 mM LOD: 0.046 mΜ	[19]
Alginate beads	GOx−HRP−TMB	B/W ^3^, R, G, B	Reaction time: 13 min Sample volume: 150 μL Linear range: 10–1000 µM LOD: 3.8 µM LOQ: 12.7 µM	[20]
Cotton fabric	GOx−HRP−TMB GOx−HRP−KI	RGB/R, G, B and CIELAB/L, a, b	Reaction time: 3 min Sample volume: 10 μL Linear range: 0.03–1 mM LOD: 0.03 mM	[37]
CNPs	GOx−HRP−ABTS	CIELAB/ΔΕ	Reaction time: 10 min Sample volume: 4.94 ± 0.25 μL Linear range: 0.025–0.375 mM LOD: 0.023 mM LOQ: 0.078 mM	This work

^1.^ TMB: 3,3′,5,5′-tetramethylbenzidine, ^2^ RGB: red, green, blue, ^3^ W/B: white/black.

**Table 3:** Performance metrics of the RANSAC algorithm for glucose prediction under different evaluation methods.

Evaluation Method	R^2^	MAE (mM)	RMSE (mM)
Test set	0.89	0.0245	0.0292
5-fold cross-validation	0.85 ± 0.02	0.0267 ± 0.0015	0.0324 ± 0.0022

**Table 4:** Recovery rate of CNP−MN biosensor.

Spiked Glucose (mM)	Predicted Glucose (mM)	Recovery Rate (%)
0.100	0.110	112 ± 3
0.200	0.180	89 ± 2
0.375	0.320	86 ± 1

## Data Availability

Data are contained within the article.

## Abbreviations

The following abbreviations are used in this manuscript:

MNs	Microneedles
CS	Chitosan
TPP	Sodium tripolyphosphate
CNPs	Chitosan nanoparticles
GOx	Glucose oxidase
HRP	Horseradish peroxidase
ABTS	2,2′-azino-bis(3-ethylbenzothiazoline-6-sulfonic acid)
H_2_O_2_	Hydrogen peroxide
EE	Encapsulation efficiency
AI	Artificial intelligence
ML	Machine learning
FDM	Fused deposition modeling
PLA	Polylactic acid
LR	Linear regression
RANSAC	Random Sample Consensus
LOD	Limit of detection
LOQ	Limit of quantification
RSD	Relative standard deviation
MAE	Mean absolute error
RMSE	Root mean squared error
